# Increased cardiovascular disease risk among adolescents and young adults with gastric cancer

**DOI:** 10.1007/s10120-024-01540-3

**Published:** 2024-07-30

**Authors:** Hea Lim Choi, Danbee Kang, Hyunsoo Kim, Juhee Cho, Keun Hye Jeon, Wonyoung Jung, Dong Wook Shin, Su-Min Jeong

**Affiliations:** 1 Department of Family Medicine/Executive Healthcare Clinic, Severance Hospital, Yonsei University College of Medicine, Seoul, Republic of Korea; 2 Department of Clinical Research Design and Evaluation, The Samsung Advanced Institute for Health Sciences & Technology (SAIHST), Sungkyunkwan University, Seoul, Republic of Korea; 3 Center for Clinical Epidemiology, Samsung Medical Center, Sungkyunkwan University, Seoul, Republic of Korea; 4 Department of Epidemiology and Medicine, Welch Center for Prevention, Epidemiology and Clinical Research, Johns Hopkins Medical Institutions, Baltimore, MD USA; 5 Department of Family Medicine, Cha Gumi Medical Center, Cha University, Gumi, Republic of Korea; 6 Division of Caridiology, Department of Medicine, Perelman School of Medicine, University of Pennsylvania, Philadelphia, USA; 7 Department of Digital Health, SAIHST, Sungkyunkwan University, Seoul, Republic of Korea; 8 Department of Family Medicine/Supportive Care Center, Samsung Medical Center, Sungkyunkwan University School of Medicine, Seoul, Republic of Korea; 9 Department of Medicine, Seoul National University College of Medicine, 1, Gwanak-ro, Gwanak-gu, Seoul, 08826 Republic of Korea; 10 Department of Family Medicine, Seoul National University Hospital, Seoul, Republic of Korea

**Keywords:** Adolescent health, Gastric cancer, Cardiovascular disease, Young adult, Young cancer patient

## Abstract

**Background:**

Previous studies have investigated cardiovascular disease (CVD) risks in cancer patients, but there is limited knowledge concerning the CVD risk in adult and young adolescent (AYA) survivors of gastric cancer.

**Objectives:**

This study aims to investigate the incidence of CVD in AYA gastric cancer survivors, analyzing it by treatment type and identifying associated risk factors.

**Methods:**

We conducted a retrospective cohort study using Korean National Health Insurance Service data collected from 2006 to 2019. Propensity score matching (1:3, caliper < 0.1) was performed using the variables age, sex, income, residential area, and presence of comorbidities, and we classified participants into gastric cancer (*n* = 6562) and non-cancer control (*n* = 19,678) groups. Cox regression models were used to calculate hazard ratios (HRs) for CVD incidence. The study assessed CVD incidence by cancer treatment and identified risk factors through multivariable Cox regression.

**Results:**

During a median 6.5-year follow-up, AYA gastric cancer survivors consistently exhibited greater CVD incidence. Their risk of CVD was significantly elevated compared to that of controls (HR, 1.18; 95% confidence interval [CI] 1.05–1.33). In particular, deep vein thrombosis (HR, 3.93; 95% CI 3.06–14.67) and pulmonary embolism (HR, 6.58; 95% CI 3.06–14.67) risks were notably increased. Chemotherapy was associated with an increased risk of stroke, heart failure, atrial fibrillation, deep vein thrombosis, and pulmonary embolism. Hypertension (HR, 1.58; 95% CI 1.10–2.26) and dyslipidemia (HR, 1.46; 95% CI 1.06–2.20) emerged as risk factors for CVD development.

**Conclusion:**

This study reports elevated risks of CVD in AYA gastric cancer survivors and emphasizes the need for vigilant monitoring of CVD in this population.

**Supplementary Information:**

The online version contains supplementary material available at 10.1007/s10120-024-01540-3.

## Introduction

Gastric cancer remains a significant global health concern, accounting for a substantial burden of cancer-related morbidity and mortality [1]. Among affected individuals, adolescents and young adults (AYAs) represent a unique subset characterized by distinctive disease patterns and management challenges [2]. AYA gastric cancer patients often present with more advanced and aggressive pathogenesis compared to older gastric cancer patients, probably due to delayed diagnosis and inherited genetic factors [3]. [4]. While investigations into the cardiovascular disease (CVD) risk among cancer patients have been conducted in various contexts [5–8], research specifically targeting AYAs with gastric cancer is notably scarce.

Previous studies exploring the CVD risk in AYA cancer patients have reported the potential implications of cancer treatments and the long-term health outcomes of the AYA population [9–11]. However, most of these investigations have primarily focused on cancers that more commonly occur in Western AYAs, such as lymphoma, thyroid, or gynecologic cancer [12–14], with limited attention paid to gastric cancer. In addition, general population-based studies have reported a decreased risk of CVDs among older gastric cancer patients. One cohort study from Korea documented a decreased risk of coronary heart disease and ischemic stroke in gastric cancer survivors after gastrectomy [15]. In parallel with this result, another study found that cardiovascular risk factors such as triglycerides, low-density lipoprotein cholesterol, and body weight were decreased in gastric cancer patients after gastrectomy [16]. However, precise information on the incidence of CVD in gastric cancer patients within the AYA population remains lacking.

With the expected poor prognosis of AYA gastric cancer patients, finding a way to diminish preventable factors, such as those associated with CVD, is in demand. Therefore, there is a compelling need for targeted research aimed at investigating the specific CVD risk level within this distinct population. The present study sought to investigate the CVD risk among AYAs with gastric cancer compared to non-cancer controls and to calculate the risk of CVD according to treatment modality.

## Methods

### Data sources

We performed a retrospective, population-based cohort study using the Korean National Health Insurance Service (K-NHIS) database. Korea has a mandatory social insurance system with insurance premiums that are determined by income level and not by health status. The K-NHIS is a single insurer that covers approximately 97% of the population, while the remaining 3% of beneficiaries are covered by the Medical Aid Program. Data on the use of medical facilities and records of prescriptions with International Statistical Classification of Diseases and Related Health Problems, 10th Revision (ICD-10), diagnosis codes are gathered by the NHIS. The K-NHIS claims database also includes information on demographics, medical treatment, procedures, prescription drugs, diagnostic codes, and hospital use. Vital status and cause of death were obtained from death certificates collected by Statistics Korea at the Ministry of Strategy and Finance of South Korea [17]. Use of the K-NHIS database was approved by the NHIS review committee.

### Definition of AYA cancer survivors

The main exposure was the incidence of cancer in participants 15–39 years old. To define incident cancer, we used a special registration code V193 in addition to the relevant ICD-10 diagnosis code. The K-NHIS has established a special copayment-reduction program to enhance health coverage and reduce the financial burden of patients with cancer. Once cancer patients are registered in the system, they pay only 5% of the total medical bill incurred for cancer-related medical care. Since enrollment in this copayment-reduction program is indicated by a special copayment-reduction code for cancer (V193) and requires a medical certificate from a physician, the cancer diagnoses included in this study are considered to be sufficiently reliable, and this method has been used in previous studies [18].

### Study population

For this study, we considered all Korean men and women aged 15–39 years enrolled between 2006 and 2019 in the K-NHIS database between 2005 and 2020. Data access was restricted by a data-share policy; we selected all patients with cancer defined by the presence of ICD-10 code C or a special copayment reduction code for cancer (V193) between 2006 and 2019 (*n* = 681,752) and fourfold the number of age- and sex-matched samples of men and women who did not develop cancer during the study period (*n* = 2,670,558). To select newly diagnosed cancer as the exposure and incident cases of CVD as the outcome, we excluded 109,269 participants with CVD (*n* = 66,317) or any cancer (*n* = 45,732) before January 1, 2006.

**Fig. 1:**
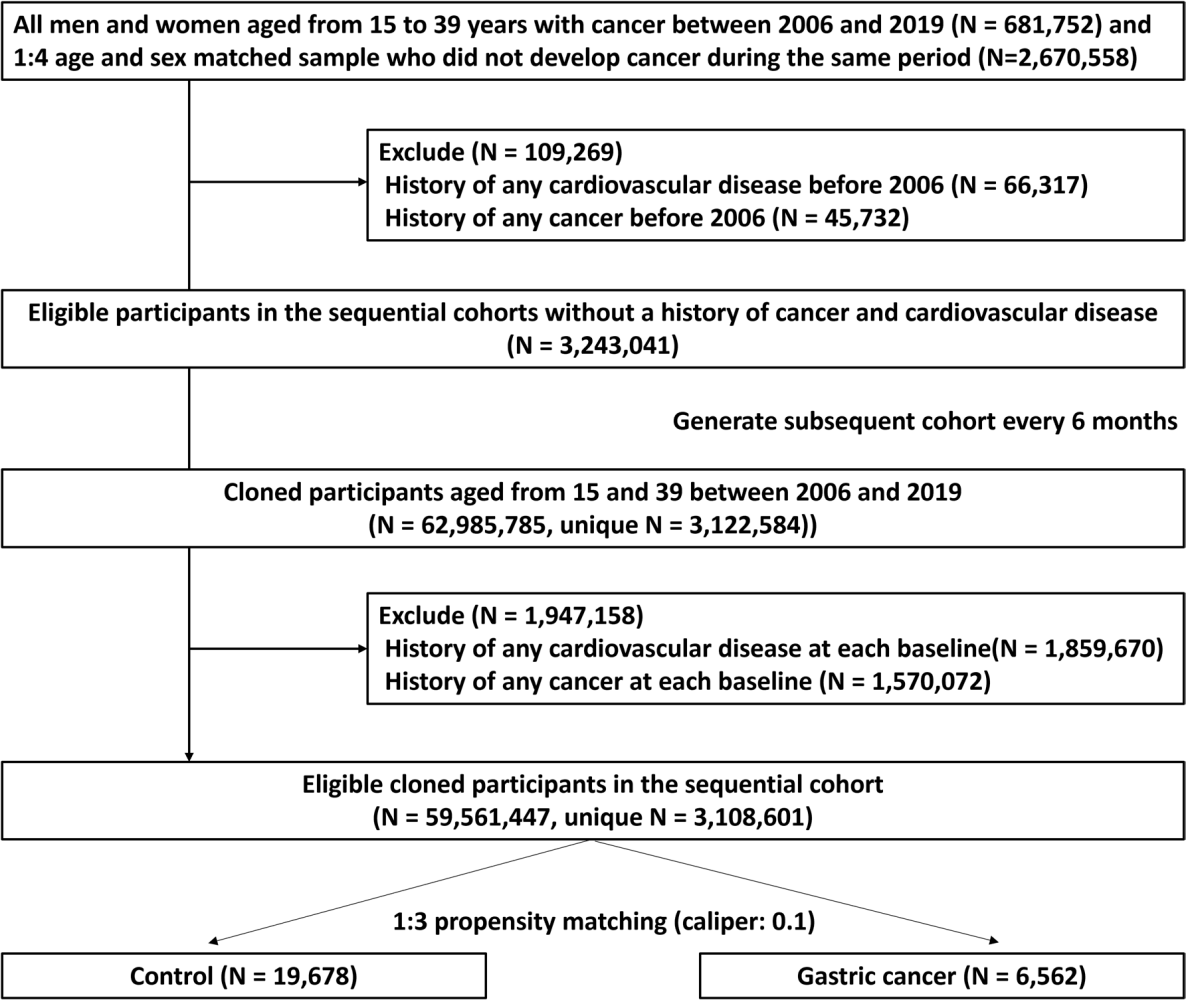
Flow chart of study population selection

Among the eligible participants (n = 3,243,041), we mimicked sequential emulation of the target trial (detailed methods are presented in the Statistical Analysis section) [19, 20]. The number of cloned participants aged 15–39 years enrolled in the K-NHIS database between 2006 and 2019 was 62,985,785. During the process of enrolling the new cohort, we excluded participants who had any cancer (*n* = 1,570,072) or a history of CVD at each baseline point (*n* = 1,859,670). Participants who met the eligibility criteria in the previous cohort were excluded from the next cohort if they were > 40 years old, had cancer or a history of CVD, or died prior to the start date. These processes were repeated every 6 months until June 1, 2019 (*n* = 59,561,447/unique *n* = 3,108,601). Since our focus was on AYA gastric cancer, patients who underwent gastric cancer surgery, including endoscopic operation of an upper gastrointestinal tumor and endoscopic submucosal dissection, as well as total and subtotal gastrectomy, within the 2 months prior to diagnosis and within 1 year thereafter with ICD-10 diagnosis code (C16) were selected for the gastric cancer group (*n* = 6562). Then, we also selected a threefold larger control group using propensity score matching (*n* = 19,678) **(**Fig. 1**).**

The Institutional Review Board of the Samsung Medical Center approved the study and waived the requirement for informed consent because K-NHIS data were de-identified (SMC 2022-03-028).

### Study outcomes

The primary endpoint is a composite outcome of any cardiac outcomes, including myocardial infarction (ICD-10: I21–I22), stroke (ICD-10: I60–I64), heart failure (ICD-10: I50), cerebrovascular disease (ICD-10: I63–I69), atrial fibrillation (ICD-10: I48), arrhythmia (ICD-10: I47–I49), cardiomyopathy (ICD-10: I42–I43, I23.5), valvular heart disease (ICD-10: I01–I08, I34–I37), venous thromboembolism (VTE): deep venous thromboembolism (DVT) (ICD-10: I80.1–I80.3), and pulmonary embolism (PE) (ICD-10: I26). The cardiovascular outcomes were identified by diagnostic records, according to the ICD-10 codes from either outpatient visits or hospitalization. The definitions of outcomes are summarized in Supplemental Table 1. In regard to myocardial infarction diagnosis, 93% accuracy was achieved in the validation study [21].

### Other variables

For the covariates, we included age, sex, comorbidities, income, and residential area at baseline. The presence of diabetes (ICD-10, E100-E149), hypertension (ICD-10, I10-I15), or dyslipidemia (ICD-10, E780-E785) was defined as having had at least one clinic visit or hospitalization with the corresponding ICD-10 code within the previous year. Data on income were obtained from the insurance eligibility database. Income level was categorized by percentile (≤ 30th, > 30th– ≤ 70th, and > 70th percentiles). Residential area was classified as metropolitan or rural. Metropolitan areas were defined as Seoul, six metropolitan cities, and 15 cities with > 5,00,000 residents that have been officially designated as municipal cities (http://www.mois.go.kr). We conducted a sensitivity analysis to examine whether body mass index (BMI), alcohol drinking, smoking, and physical activity have an additional impact on CVD outcome. For this analysis, we restricted the participants to those who underwent a health screening exam 4 years prior to baseline.

### Statistical analysis

The participants were then classified into two groups based on whether they developed gastric cancer or not. In this process, we generated a propensity score using logistic regression with incident cancer as the outcome variable and age, sex, income, residential area, and the presence of comorbidities (diabetes mellitus, hypertension, and hyperlipidemia) at cohort entry as covariates. Then, a 1:3 matching ratio was applied using the propensity score through greedy matching methods (caliper < 0.1). To compare the distribution of variables used for matching, a standardized mean difference between the gastric cancer and control groups was estimated. The variables used for matching were updated based on the first date for each subsequent cohort.

The primary endpoint was development of CVD. Each endpoint was analyzed separately, and we included only participants who had not experienced the endpoint of interest prior to the endpoint analysis. We followed the participants from the baseline of each subsequent cohort until the development of CVD, death, or December 2020, whichever occurred first. After completing all processes, we pooled the data from all trials into a single model and included the day at baseline of each cohort.

The cumulative incidence of each outcome was estimated with the Kaplan–Meier method, and log-rank tests were applied to evaluate differences between the groups. We calculated hazard ratios (HR) with 95% confidence intervals (CIs) for clinical outcome incidence using a Cox regression model. The time scale was the calendar year. We examined the proportional hazards assumption using plots of the log (-log) survival function and Schoenfeld residuals.

In sensitivity analysis, to account for competing risks due to mortality, we fitted a proportional subdistribution hazards (subHR) regression model [22] with death as the competing event. Since the year 2020 was complicated by the coronavirus disease 2019 pandemic, we performed a separate sensitivity analysis to exclude the year 2020. To understand the differential impacts of treatment modalities on the outcomes of interest, additional subgroup analyses were performed in the surgery-only group and the chemotherapy group. These groups were compared to their matched controls from the general population without gastric cancer at baseline. In addition, individual control group tests were conducted for each subgroup. We also performed multivariable Cox regression to find risk factors influencing the incidence of CVD among AYA gastric cancer patients after adjusting for covariates of BMI, smoking status, alcohol consumption and regular physical activity. As the risk of VTE significantly increased in AYA gastric cancer patients, we also presented risk factors affecting the incidence of VTE.

All *P* values were two-sided, and *P* < 0.05 was considered statistically significant. Analyses were performed with SAS® Visual Analytics (SAS Institute Inc., Cary, NC, USA) and R 4.1.2 (R Foundation for Statistical Computing, Vienna, Austria).

## Results

### Baseline characteristics

The mean age of study participants was 35 years, and 44% of participants were men (Table 1). All standardized mean differences between the control and AYA gastric cancer groups were < 0.1. Most AYA gastric cancer patients received only surgery (64.8%), followed by surgery with chemotherapy (27.3%).

**Table 1:** Baseline characteristics of study population

	Control	AYA gastric cancer	SMD
	*n* = 19,678	*n* = 6562	
Age, years (mean (SD))	34.9 (4.0)	35.0 (3.8)	0.016
Sex (%)			0.006
Male	8699 (44.2)	2919 (44.5)	
Female	10,979 (55.8)	3643 (55.5)	
Income (%)			
Medical aid	202 (1.0)	55 (0.8)	−0.020
≤ 30th	380 (19.7)	1305 (19.9)	0.004
31st–70th	8518 (43.3)	2867 (43.7)	0.008
> 70th	6635 (33.7)	2183 (33.3)	−0.010
Residential area, metropolitan (%)	13,295 (67.6)	4416 (67.3)	−0.006
Comorbidities			
Diabetes, yes (%)	664 (3.4)	223 (3.4)	0.001
Hypertension, yes (%)	1177 (6.0)	398 (6.1)	0.004
Hyperlipidemia, yes (%)	1754 (8.9)	593 (9.0)	0.004
Type of treatment (%)			
Surgery	–	4254 (64.8)	–
Surgery + CTx	–	1789 (27.3)	–
Surgery + RT	–	27 (0.4)	–
Surgery + CTx + RT	–	178 (2.7)	–
ESD only	–	314 (4.8)	–
ESD to surgery (%)	–	66 (1.0)	–

*AYA* adolescent and young adult, *CTx* chemotherapy, *ESD* endoscopic submucosal dissection, *RT* radiation therapy, *SMD* standard mean difference

### Difference in CVD risk between AYA gastric cancer patients and matched controls

**Fig. 2:**
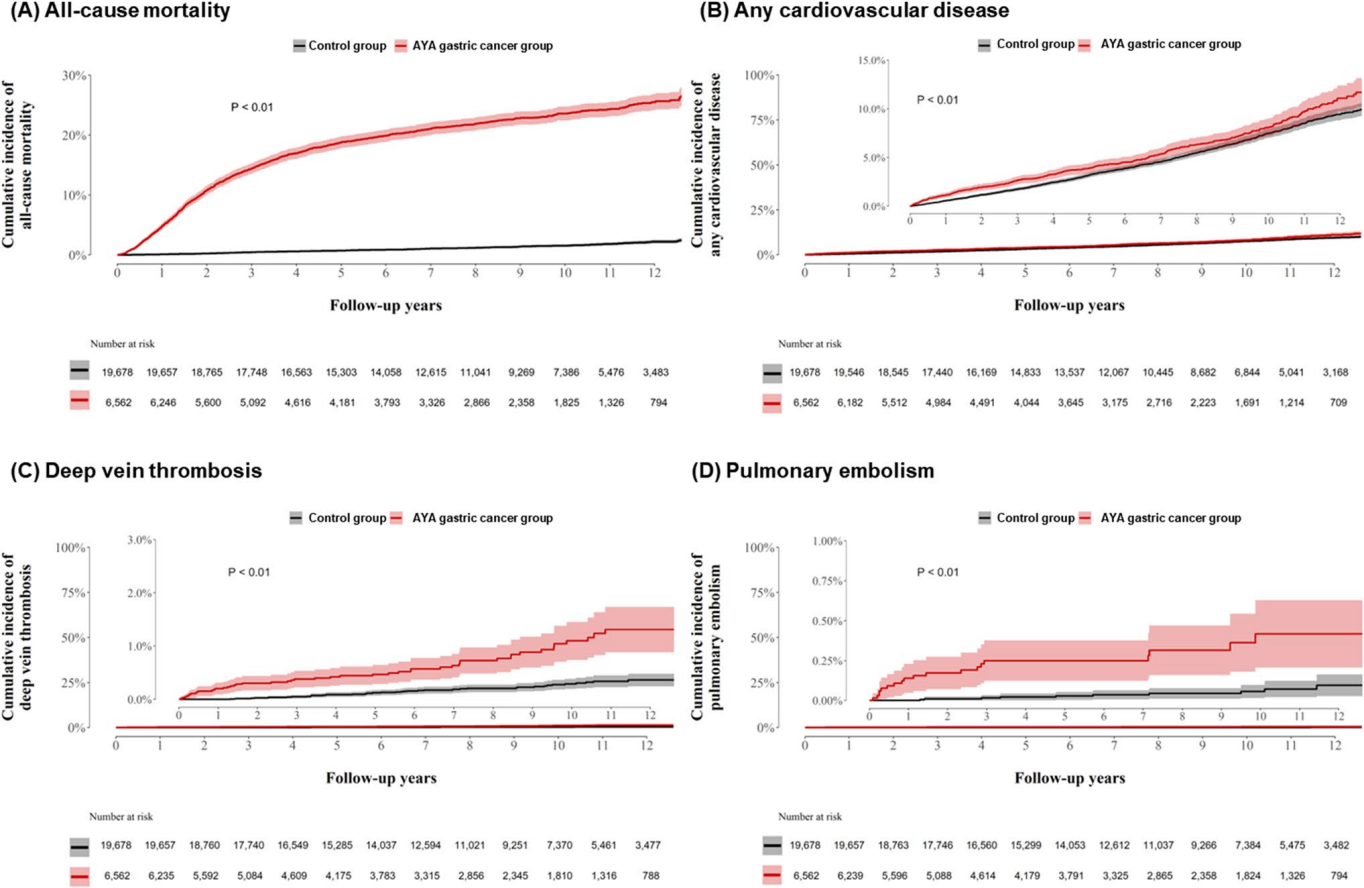
Cumulative incidence of all-cause mortality, any CVD, DVT, and PE between AYA gastric cancer patients and controls

During follow-up (median, 6.5 years), 1535 participants developed CVD **(**Table 2**)**. The cumulative incidence of CVD was consistently higher in participants with gastric cancer compared to those without gastric cancer during the entire follow-up period **(**Fig. 2B**)**. The HR for CVD when comparing AYA gastric cancer patients to controls was 1.18 (95% CI 1.05–1.33). However, the association became no significant after competing risk analysis (subHR, 1.00; 95% CI 0.89–1.12). There were no significant differences in BMI, smoking status, drinking status, and physical activity between AYA cancer survivors and controls (Supplemental Table 2). After adjusting these lifestyle covariates, the results were consistent with main findings (Supplemental Table 3**).**

**Table 2:** HR (95% CI) values for incident CVD associated with AYA gastric cancer

	No. of cases (100 person-years)		HR (95% CI)^a^	SubHR (95% CI)^b^
	Control	Cancer		
All-cause death	271 (0.2)	1380 (3.1)	**17.78 (15.60–20.26)**	
Any cardiovascular disease	1157 (0.8)	378 (0.9)	**1.18 (1.05–1.33)**	1.00 (0.89–1.12)
Ischemic heart disease (I20–I25)	422 (0.3)	95 (0.2)	0.81 (0.65–1.01)	0.68 (0.55–0.85)
Myocardial infarction (I21–22)	30 (0.0)	3 (0.0)	0.37 (0.11–1.22)	0.31 (0.09–1.01)
Cerebrovascular disease (I63–I69)	346 (0.2)	96 (0.2)	1.01 (0.81–1.27)	0.85 (0.67–1.06)
Stroke (I60–I64)	132 (0.1)	39 (0.1)	1.06 (0.74–1.52)	0.90 (0.63–1.29)
Ischemic stroke (I60–I62)	89 (0.1)	26 (0.1)	1.05 (0.68–1.62)	0.89 (0.58–1.38)
Hemorrhagic stroke (I63–I64)	49 (0.0)	17 (0.0)	1.26 (0.73–2.19)	1.06 (0.61–1.83)
Heart failure (I50)	101 (0.1)	40 (0.1)	1.44 (0.99–2.08)	1.20 (0.83–1.73)
Cardiomyopathy (I42–I43, I23.5)	22 (0.0)	6 (0.0)	1.02 (0.41–2.51)	0.82 (0.33–2.03)
Valvular heart disease (I01–I08, I34–I37)	13 (0.0)	3 (0.0)	0.83 (0.24–2.93)	0.70 (0.20–2.44)
Arrhythmia (I47–I49)	393 (0.3)	132 (0.3)	1.21 (0.99–1.48)	1.02 (0.84–1.24)
Atrial fibrillation (I48)	67 (0.0)	20 (0.0)	1.10 (0.67–1.81)	0.91 (0.55–1.50)
Venous thromboembolism				
Deep vein thrombosis (I80.1–I80.3)	43 (0.0)	47 (0.1)	**3.93 (2.60–5.95)**	**3.32 (2.20–5.02)**
Pulmonary embolism (I26)	10 (0.0)	19 (0.0)	**6.58 (3.06–14.67)**	**5.75 (2.68–12.35)**

Bold font indicates statistical significance (*p* < 0.05)

*AYA* adolescent and young adult, *CI* confidence interval, *HR* hazard ratio

^a^Matching variables: age, sex, income, residential area, and comorbidities (diabetes mellitus, hypertension, and hyperlipidemia)

^b^Subhazard ratios for events were modeled with mortality as a competing risk

When stratified by specific outcomes, there was no significant difference in ischemic heart disease, cerebrovascular disease, heart failure, valvular heart disease, arrhythmia, cardiomyopathy, or valvular heart disease between the AYA gastric cancer patients and the control group. The cumulative incidence rates of DVT and PE were consistently higher in AYA gastric cancer patients compared to individuals without gastric cancer during the entire follow-up period **(**Fig. 2 C, D**)**. The HRs for DVT and PE, when comparing AYA gastric cancer patients to individuals without gastric cancer, were 3.93 (95% CI 3.06–14.67) and 6.58 (95% CI 3.06–14.67), respectively. This association remained significant in competing risk analyses. In the sensitivity analysis using data recorded until 2019, the trends were similar (Supplemental Table 4).

### Subgroup analysis by chemotherapy

In the subgroup analysis, patients who received chemotherapy showed a greater risk of CVD (HR, 1.60; 95% CI 1.30–1.96) **(**Table 3**)**. Compare to the control group, the AYA gastric cancer group receiving chemotherapy also showed a significant increase in stroke (HR, 2.33; 95% CI 1.21–4.26), heart failure (HR, 2.38; 95% CI 2.30–4.34), atrial fibrillation (HR, 2.31; 95% CI 1.11–4.82), DVT (HR, 10.33; 95% CI 6.28–16.99), and PE (HR, 23.71; 95% CI 10.42–53.92), but not myocardial infarction, valvular heart disease, arrhythmia, or cardiomyopathy.

**Table 3:** Subgroup analysis by receipt of chemotherapy

	No. of cases (100 person-years) of	HR (95% CI)^a^	SubHR (95% CI)^b^
	AYA gastric cancer		
**Surgery without chemotherapy**			
Any cardiovascular disease	241 (0.8)	1.03 (0.89–1.18)	0.99 (0.86–1.14)
Ischemic heart disease	70 (0.2)	0.81 (0.63–1.04)	0.78 (0.61–1.01)
Myocardial infraction	3 (0)	0.51 (0.15–1.66)	0.48 (0.15–1.58)
Cerebrovascular disease	64 (0.2)	0.92 (0.70–1.20)	0.88 (0.67–1.15)
Stroke	18 (0.1)	0.68 (0.41–1.11)	0.65 (0.40–1.06)
Ischemic stroke	11 (0)	0.61 (0.33–1.15)	0.59 (0.32–1.10)
Hemorrhagic stroke	8 (0)	0.81 (0.38–1.72)	0.77 (0.37–1.63)
Heart failure	25 (0.1)	1.21 (0.78–1.88)	1.17 (0.75–1.81)
Cardiomyopathy	5 (0)	1.13 (0.43–3.00)	1.06 (0.40–2.80)
Valvular heart disease	1 (0)	0.40 (0.05–3.03)	0.36 (0.05–2.73)
Arrhythmia	94 (0.3)	1.18 (0.94–1.47)	1.13 (0.90–1.42)
Arterial fibrillation	10 (0)	0.74 (0.38–1.44)	0.71 (0.36–1.39)
Venous thromboembolism			
Deep vein thrombosis	18 (0.1)	**2.08 (1.20–3.61)**	**1.97 (1.13–3.41)**
Pulmonary embolism	3 (0)	1.51 (0.41–5.50)	1.42 (0.39–5.14)
**Surgery with chemotherapy**			
Any cardiovascular disease	99 (1.1)	**1.60 (1.30–1.96)**	0.91 (0.74–1.11)
Ischemic heart disease	18 (0.2)	0.81 (0.50–1.29)	0.45 (0.28–0.72)
Myocardial infraction	0 (0)	–	–
Cerebrovascular disease	19 (0.2)	1.02 (0.64–1.63)	0.58 (0.36–0.91)
Stroke	17 (0.2)	**2.33 (1.40–3.87)**	1.36 (0.82–2.26)
Ischemic stroke	11 (0.1)	**2.27 (1.21–4.26)**	1.30 (0.69–2.44)
Hemorrhagic stroke	8 (0.1)	**2.91 (1.37–6.18)**	1.73 (0.82–3.66)
Heart failure	12 (0.1)	**2.38 (1.30–4.34)**	1.26 (0.69–2.30)
Cardiomyopathy	1 (0)	0.93 (0.12–6.92)	0.48 (0.07–3.61)
Valvular heart disease	1 (0)	1.57 (0.20–12.20)	0.82 (0.11–6.29)
Arrhythmia	28 (0.3)	1.32 (0.90–1.94)	0.75 (0.51–1.11)
Arterial fibrillation	8 (0.1)	**2.31 (1.11–4.82)**	1.26 (0.61–2.63)
Venous thromboembolism			
Deep vein thrombosis	25 (0.3)	**10.33 (6.28–16.99)**	**6.25 (3.81–10.26)**
Pulmonary embolism	14 (0.2)	**23.71 (10.42–53.92)**	**15.10 (6.67–34.18)**

Bold font indicates statistical significance (*p* < 0.05)

*CI* confidence interval, *HR* hazard ratio

^a^Matching variables: age, sex, income, residential area, and comorbidities (diabetes mellitus, hypertension, and hyperlipidemia)

^b^Subhazard ratios for events were modeled with mortality as a competing risk

The AYA gastric cancer group without chemotherapy did not show a significant difference in CVD risk compared to the control group (HR, 0.99; 95% CI 0.86–1.14). However, the risk of DVT remained high in the AYA gastric cancer group without chemotherapy compared to the control group (HR, 1.97; 95% CI 1.13–3.41). The results were similar when we selected individual matched group within each subgroup (Supplemental Table 5).

### Factors associated with CVD and VTE

Among AYA gastric cancer patients, those with hypertension (HR, 1.58; 95% CI 1.10–2.27) or hyperlipidemia (HR, 1.46; 95% CI 1.06–2.20) had a greater risk of CVD incidence **(**Table 4**)**. Patients with chemotherapy also had a greater risk of VTE incidence, albeit without statistical significance (HR, 5.11; 95% CI 0.69–38.04).

**Table 4:** Risk factors of CVD and venous thromboembolism in AYA gastric cancer patients

	Any cardiovascular disease		Venous thromboembolism	
	No. of cases (100 person-years)	Adjusted HR^a^ (95% CI)	No. of cases (100 person-years)	Adjusted HR^a^ (95% CI)
Age at diagnosis (years)		1.02 (0.99–1.05)		1.05 (0.96–1.15)
Sex, female				
Male	171 (0.9)	Reference	20 (0.1)	Reference
Female	207 (0.9)	1.00 (0.81–1.23)	27 (0.1)	1.06 (0.59–1.91)
Income				
Medical aid	5 (1.5)	1.75 (0.71–4.29)	0 (0)	-
≤ 30th	89 (1.1)	1.21 (0.92–1.59)	12 (0.1)	1.42 (0.65–3.10)
31st–70th	147 (0.8)	0.91 (0.71–1.16)	20 (0.1)	1.12 (0.56–2.24)
> 70th	132 (0.9)	Reference	14 (0.1)	Referenc*e*
Residential area				
Rural	125 (1.0)	Reference	10 (0.1)	Reference
Metropolitan	248 (0.9)	0.90 (0.73–1.13)	36 (0.1)	1.68 (0.83–3.39)
Type of treatment				
ESD only	17 (0.9)	Reference	1 (0.1)	Reference
Surgery only	241 (0.8)	0.82 (0.50–1.35)	18 (0.1)	1.02 (0.14–7.65)
Surgery with chemotherapy	99 (1.1)	1.19 (0.71–2.01)	25 (0.3)	5.11 (0.69–38.04)
Others	21 (1.9)	**1.95 (1.02–3.70)**	3 (0.3)	4.30 (0.44–41.58)
Comorbidities				
Diabetes				
No	363 (0.9)	Reference	45 (0.1)	Reference
Yes	15 (1.0)	1.02 (0.60–1.71)	2 (0.1)	1.52 (0.36–6.41)
Hypertension				
No	345 (0.9)	Reference	43 (0.1)	Reference
Yes	33 (1.4)	**1.58 (1.10–2.27)**	4 (0.2)	1.76 (0.62–5.01)
Hyperlipidemia				
No	334 (0.8)	Reference	44 (0.1)	Reference
Yes	45 (1.3)	**1.46 (1.06–2.20)**	3 (0.1)	0.72 (0.22–2.40)

Bold font indicates statistical significance (*p* < 0.05)

*AYA* adolescent young adult, *CI* confidence interval, *ESD* endoscopic submucosal dissection, *HR* hazard ratio

^a^Matching variables: age, sex, income, residential area, and comorbidities (diabetes mellitus, hypertension, and hyperlipidemia)

## Discussion

This large population-based cohort study revealed an increased risk of CVD in AYAs with gastric cancer, including a significant increase in both DVT and PE. This increased risk of CVD was more prominent in AYA gastric cancer patients who underwent chemotherapy, while the risk of DVT was high regardless of chemotherapy. In addition, we not only examined the CVD risk of AYA gastric cancer patients but also identified the risk factors associated with an increased CVD risk. To the best of our knowledge, the specific incidence of CVD in AYAs with gastric cancer alone has not been reported previously [11, 12, 23], making this the first study to investigate this incidence. Previous studies have either excluded gastric cancer from investigations into the most common cancers [4] largely due to its lower incidence in Western populations and AYAs or have included it as a gastrointestinal tract cancers.

Contrary to AYA gastric cancer, a study that enrolled older gastric patients reported that gastric cancer was associated with decreased risk of coronary heart disease and ischemic stroke [15]. This may be attributed to the weight loss associated with the reduced stomach capacity after gastrectomy, leading to improvements in metabolic risk factors related to CVD [16, 24]. However, the favorable changes in lipid profile diminished as time passed after surgery [24]. From this result, we can infer that the immediate favorable effect of gastrectomy on metabolic risk factors could be diminished in AYA gastric cancer patients compared to older patients as time passes over a longer period of survivorship. In addition, since the prevalence of known CVD risk factors such as obesity [25] and metabolic syndrome [26] is high among older patients compared to young individuals, the positive outcome from gastrectomy may only be prominent among older gastric cancer patients and not AYA gastric cancer patients.

AYA cancer patients are not eligible for national cancer screening programs. In Korea, the biannual health check-up, which includes upper endoscopy, is recommended for individuals aged ≥ 40 years [15]. Therefore, unless there are specific symptoms, it is not easy to detect gastric cancer at an early stage among young individuals. As a result, AYA gastric cancer cases often present in an advanced stage [3, 27]. In addition, the histological types of cancer observed in AYA gastric cancer patients are often poorly differentiated or of the diffuse type [3, 4, 27], and these cases are known to be more aggressive compared to those in adults, leading to a reported poor prognosis. The location of gastric cancer is also frequently observed in the middle third or entirety of the stomach [4]. Another characteristic of AYA gastric cancer is a female predominance [3, 4, 27], which was consistently observed in our study.

In our study, the risk of overall CVD in AYA gastric cancer patients who received chemotherapy was 1.6 times that of the matched controls. Since younger gastric cancer patients are diagnosed relatively late and show more aggressive biological cancer behaviors, as previously mentioned, they are more likely to undergo additional chemotherapy rather than surgery alone. Previous population-based studies have reported that gastric cancer patients have a higher CVD mortality rate than that of non-cancer individuals, with a relative risk ranging from 1.13–3.93 [5, 6, 28]. In particular, younger age at cancer diagnosis is associated with increased CVD mortality [6], which can be explained by cumulative cardiac damage due to prolonged exposure to chemotherapy [10, 13] or radiotherapy [28]. Consequently, it is speculated that younger age at chemotherapy initiation leads to longer exposure to chemotherapy, contributing to more frequent development of CVD.

Notably, in this study, we demonstrated a significantly increased risk for VTE in AYA gastric cancer patients. The risk of PE was increased 15.10-fold and that of DVT was increased 6.25-fold in those who underwent surgery and chemotherapy. Moreover, a sustained increased risk for DVT and PE was observed in AYA gastric cancer patients throughout the study follow-up period.

Our results are concurrent with those of previous studies reporting an increased VTE risk among gastric cancer patients [29, 30]. Gastric cancer is reported to be one of the common solid tumors with the highest risk of VTE [31], and gastric cancer patients have been reported to experience more cancer-associated thrombotic events [32]. Cancer initiates thrombotic events by producing tissue factors and cancer procoagulants, which lead to amplification of the coagulation cascade [33]. Therefore, an increased risk of VTE was speculated in AYA gastric cancer survivors compared to a non-cancer control group due to increased platelet activation and the formation of thrombi. An increase in venous stasis caused by the surgical procedure itself or postoperative performance status and immobility could be risk factor for VTE as well [34], accounting for the 2.08-fold increased risk of DVT in AYA gastric cancer patients who only received surgery in our study. Most of all, previous studies demonstrated an increased risk of VTE in patients with advanced and metastatic gastric cancer, who are more likely to undergo chemotherapy [35–37]. A study conducted in Korea investigated the incidence of VTE in advanced gastric cancer patients and found that patients with inoperable gastric cancer, who were most likely to receive anticancer treatment, had a high risk of developing VTE [38], with a 1-year cumulative incidence rate of VTE of 3.5% documented in advanced inoperable gastric cancer patients. Meanwhile, another study of Japanese gastric cancer patients reported an incidence of VTE of 18% in metastatic stomach cancer patients receiving chemotherapy [39]. Here, we observed the risk of VTE in AYA gastric cancer to be higher than what has been reported in other studies. This could be attributed to the fact that AYA gastric cancer patients undergo prolonged exposure to chemotherapy, and a young-aged non-cancer control group is less likely to experience VTE events.

Our study revealed a significant increase in the risk of CVD among AYA gastric cancer survivors, especially those who received chemotherapy and had comorbidities such as hypertension and dyslipidemia. Therefore, AYA gastric cancer survivors who receive chemotherapy and have comorbidities require more medical attention during surveillance periods.

Our study also confirms a meaningful increase in the risk of VTE in AYA gastric cancer survivors regardless of chemotherapy. It is suggested that VTE is associated with decreased life expectancy in cancer patients [40], so recognizing its risk and preventing it adequately by providing thromboprophylaxis to AYA gastric cancer survivors would relieve additional complications and burdens. Moreover, further investigation is warranted to elucidate the underlying mechanism of increased VTE among AYA gastric cancer survivors.

### Study limitations

Despite the strength of enrolling a large, unselected Korean population, this study has several limitations. First, due to the use of administrative data, we lacked detailed clinicopathological information, such as cancer stage and histology. However, we could infer such details based on the information about the treatments received by the patients. Second, the use of claims data can involve inaccurate outcome definitions, so the actual incidence could be either underrated or exaggerated. Finally, this study focused on Korean individuals, whose AYA cancer incidence may differ from that of Western populations, so generalizing the results might be challenging.

## Conclusion

In conclusion, this large population-based cohort study confirmed a potential risk for CVD, especially VTE, in AYA gastric cancer survivors. This risk was prominently high among patients who had received chemotherapy. Our results highlight the importance of closely monitoring AYA gastric cancer survivors to enhance their prognosis by proactively mitigating the incidence of CVD, especially including preventing VTE events by providing prompt prophylactic treatment. Further research is warranted to elucidate the underlying mechanisms contributing to this increased risk.

## Supplementary Information

Below is the link to the electronic supplementary material.

Supplementary file1 (DOCX 40 KB)
