# Supplementary material for: Increased cardiovascular disease risk among adolescents and young adults with gastric cancer
Source: Gastric Cancer. 2024 Jul 30;27(6):1169–79. doi: 10.1007/s10120-024-01540-3 (PMC11513758; doi:10.1007/s10120-024-01540-3)
Supplement: Supplementary file 1 — Supplementary file1 (DOCX 40 KB) [file 10120_2024_1540_MOESM1_ESM.docx]

**Supplemental Table 1. Definition of outcomes**

| **Outcome** | **ICD 10 code or**  **Procedure code** | **Diagnostic definition** |
| --- | --- | --- |
| **Gastric cancer** |  |  |
| Total gastrectomy | Q2533, Q2536, Q2534, Q2537 |  |
| Subtotal gastrectomy | Q2594, Q0251, Q0252, Q0253, Q0254, Q0255, Q0256, Q0257, Q0258, Q2598 |  |
| Endoscopic operation of upper gastrointestinal tumor | Q7651, Q7652 |  |
| Endoscopic submucosal dissection | Q7653, Q7654 |  |
| **Cardiovascular disease** |  |  |
| **Ischemic heart disease** | I20–I25 (primary and 1^st^ sub) | Admission or outpatient department ≥ 1 |
| Myocardial infraction | I21–I22 (primary and 1^st^ sub) | Admission or outpatient department ≥ 1 |
| **Cerebrovascular disease** | I63–I69 (primary and 1^st^ sub) | Admission or outpatient department ≥ 1 |
| **Stroke** | I60–I64 (primary and 1^st^ sub) | Admission or outpatient department ≥ 1 |
| Hemorrhagic stroke | I60–I62 (primary and 1^st^ sub) | Admission or outpatient department ≥ 1 |
| Ischemic stroke | I63–I64 (primary and 1^st^ sub) | Admission or outpatient department ≥ 1 |
| **Heart failure** | I50 (primary and 1^st^ sub) | Admission or outpatient department ≥ 1 |
| Cardiomyopathy | I42–I43, I23.5 | Admission or outpatient department ≥ 1 |
| Valvular heart disease | I01–I08, I34–I37 | Admission or outpatient department ≥ 1 |
| **Arrhythmia** | I47–I49 | Admission or outpatient department ≥ 1 |
| Atrial fibrillation | I48 | Admission or outpatient department ≥ 1 |
| **Venous thromboembolism** |  |  |
| Deep venous thromboembolism | I80.1–I80.3 | Admission or outpatient department ≥ 1 |
| Pulmonary embolism | I26 | Admission or outpatient department ≥ 1 |

**Supplemental Table 2.** **Characteristic of study participants who had received health screening examination (N = 16,208)**

|  | **Control** | **AYA gastric Cancer** | **SMD** |
| --- | --- | --- | --- |
|  | n = 12,329 | n = 3,879 |  |
| **Age, years (mean (SD))** | 35.3 (3.6) | 35.2 (3.6) | -0.032 |
| **Sex (%)** |  |  | 0.041 |
| Male | 6,170 (50.0) | 2,021 (52.1) |  |
| Female | 6,159 (50.0) | 1,858 (47.9) |  |
| **Income (%)** |  |  |  |
| Medical Aid | 85 (0.7) | 16 (0.4) | -0.037 |
| ≤30^th^ | 2,232 (18.1) | 685 (17.7) | -0.012 |
| 31^st^–70^th^ | 5,651 (45.8) | 1,772 (45.7) | -0.003 |
| >70^th^ | 4,111 (33.3) | 1,285 (33.1) | -0.005 |
| **Residential area, metropolitan (%)** | 8,403 (68.2) | 2,601 (67.1) | -0.024 |
| **Comorbidities** |  |  |  |
| Diabetes, yes (%) | 412 (3.3) | 128 (3.3) | -0.002 |
| Hypertension, yes (%) | 713 (5.8) | 231 (6.0) | 0.007 |
| Hyperlipidemia, yes (%) | 1,222 (9.9) | 368 (9.5) | -0.014 |
| **Type of treatment (%)** |  |  |  |
| Surgery | - | 2,666 (68.7) | - |
| Surgery + CTx | - | 871 (22.5) | - |
| Surgery + RT | - | 20 (0.5) | - |
| Surgery + CTx + RT | - | 93 (2.4) | - |
| ESD only | - | 229 (5.9) | - |
| **ESD to surgery (%)** | - | 47 (1.2) | - |
| **Smoking status (%)** |  |  |  |
| Never | 7,437 (60.3) | 2,228 (57,4) | -0.059 |
| Former | 1,177 (9.5) | 442 (11.4) | 0.060 |
| Current | 3,582 (29.1) | 1,167 (30.1) | 0.023 |
| Unknown | 133 (1.1) | 42 (1.1) | 0 |
| **Drinking status (%)** |  |  |  |
| Never | 4,746 (38.5) | 1,620 (41.8) | 0.067 |
| Mild | 6,387 (51.8) | 1,919 (49.5) | -0.047 |
| Moderate | 350 (2.8) | 93 (2.4) | -0.028 |
| Heavy | 3 (0.0) | 0 (0.0) | -0.022 |
| Unknown | 843 (6.8) | 247 (6.4) | -0.019 |
| **Regular physical activity, yes (%)** | 1,719 (13.9) | 575 (14.8) | 0.025 |
| **BMI (km/m^2^), (mean (SD))** | 23.2 (3.7) | 22.6 (3.6) | -0.170 |
| Underweight (< 18.5) | 822 (6.7) | 423 (10.9) | 0.150 |
| Normal (18.5 – 23.0) | 5,739 (46.5) | 1,897 (48.9) | 0.047 |
| Overweight (23.0 – 25.0) | 2,315 (18.8) | 679 (17.5) | -0.033 |
| Obese (≥ 25.0) | 3,451 (28.0) | 879 (22.7) | -0.123 |
| Unknown | 2 (0.0) | 1 (0.0) | 0.007 |

AYA, adolescent and young adult; CTx, chemotherapy; RT, radiotherapy; ESD, endoscopic submucosal dissection; BMI, body mass index; SD, standard deviation; SMD, standardized mean difference.

**Supplemental Table 3. HR (95% CI) values for incident CVD associated with AYA gastric cancer among participants who had received health screening examination after further adjusting lifestyle covariates. (N = 16,208)**

|  | **No. of cases**  **(100 person-years)** | | **Adjusted HR (95% CI)*** |
| --- | --- | --- | --- |
|  | **Control** | **Cancer** |  |
| **All-cause death** | 116 (0.1) | 589 (2.2) | **18.09 (14.83, 22.09)** |
| **Any cardiovascular disease** | 721 (0.8) | 210 (0.8) | 1.11 (0.95, 1.29) |
| **Ischemic heart disease (I20–I25)** | 265 (0.3) | 54 (0.2) | 0.78 (0.58, 1.05) |
| Myocardial infarction (I21–22) | 16 (0) | 1 (0) | 0.28 (0.04, 2.11) |
| **Cerebrovascular disease (I63–I69)** | 210 (0.2) | 53 (0.2) | 0.98 (0.72, 1.32) |
| **Stroke (I60–I64)** | 81 (0.1) | 21 (0.1) | 0.99 (061, 1.61) |
| Ischemic stroke (I60–I62) | 54 (0.1) | 14 (0.1) | 0.96 (0.53, 1.74) |
| Hemorrhagic stroke (I63–I64) | 30 (0) | 10 (0) | 1.33 (0.65, 2.74) |
| **Heart failure (I50)** | 66 (0.1) | 22 (0.1) | 1.26 (0.77, 2.04) |
| Cardiomyopathy (I42–I43, I23.5) | 12 (0) | 4 (0) | 1.40 (0.45, 4.40) |
| Valvular heart disease (I01–I08, I34–I37) | 9 (0) | 3 (0) | 1,17 (0.32, 4.31) |
| **Arrhythmia (I47–I49)** | 239 (0.2) | 70 (0.3) | 1.08 (0.83, 1.42) |
| Atrial fibrillation (I48) | 43 (0) | 25 (0.1) | 0.83 (0.41, 1.65) |
| **Venous thromboembolism** |  |  |  |
| Deep vein thrombosis (I80.1–I80.3) | 27 (0) | 25 (0.1) | **3.72 (2.15, 6.45)** |
| Pulmonary embolism (I26) | 2 (0) | 8 (0) | **16.22 (3.38, 77.84)** |

* All the covariates (age, sex, income, residential area, comorbidities) were matched and body mass index, smoking status, alcohol consumption and regular physical activity were further adjusted.

AYA, adolescent and young adult; CI, confidence interval; HR, hazard ratio.

**Supplemental Table 4. Sensitivity analysis to exclude year 2020**

|  | **No. of cases**  **(100 person-years)** | | **Adjusted HR**  **(95% CI)*** |
| --- | --- | --- | --- |
|  | **Control** | **AYA gastric cancer** |  |
| **All-cause death** | 205 (0.1) | 1,211 (3.1) | **20.10 (17,33, 23.31)** |
| **Any cardiovascular disease** | 908 (0.7) | 334 (0.8) | **1.20 (1.06, 1.36)** |
| **Ischemic heart disease (I20–I25)** | 369 (0.2) | 84 (0.2) | 0.81 (0.64, 1.03) |
| Myocardial infarction (I21–22) | 24 (0) | 2 (0) | 0.30 (0.07, 1.29) |
| **Cerebrovascular disease (I63–I69)** | 287 (0.2) | 89 (0.2) | 1.11 (0.88, 1.74) |
| **Stroke (I60–I64)** | 114 (0.1) | 39 (0.1) | 1.22 (0.85, 1.74) |
| Ischemic stroke (I60–I62) | 79 (0.1) | 25 (0.1) | 1.11 (0.71, 1.75) |
| Hemorrhagic stroke (I63–I64) | 40 (0) | 17 (0) | 1.53 (0.87, 2.70) |
| **Heart failure (I50)** | 81 (0.1) | 34 (0.1) | **1.51 (1.01, 2.26)** |
| Cardiomyopathy (I42–I43, I23.5) | 18 (0) | 6 (0) | 1.22 (0.48, 3.08) |
| Valvular heart disease (I01–I08, I34–I37) | 12 (0) | 3 (0) | 0.90 (0.25, 3.18) |
| **Arrhythmia (I47–I49)** | 352 (0.2) | 106 (0.2) | 1.07 (0.86, 1.33) |
| Atrial fibrillation (I48) | 60 (0) | 14 (0) | 0.84 (0.47, 1.51) |
| **Venous thromboembolism** |  |  |  |
| Deep vein thrombosis (I80.1–I80.3) | 32 (0) | 44 (0.1) | **4.90 (3.10, 7.73)** |
| Pulmonary embolism (I26) | 8 (0) | 17 (0) | **7.30 (3.15, 16.94)** |

* All the covariates (age, sex, income, residential area, comorbidities) were matched and body mass index, smoking status, alcohol consumption and regular physical activity were further adjusted.

AYA, adolescent and young adult; CI, confidence interval; HR, hazard ratio.

**Supplemental Table 5. Subgroup analysis using individual matched cohort**

|  | **No. of cases**  **(100 person-years) of** | | **HR (95% CI)*** | **SubHR** **(95% CI)**† |
| --- | --- | --- | --- | --- |
|  | **Control** | **AYA gastric cancer** |  |  |
| **Surgery without chemotherapy (N = 17,009)** |  |  |  |  |
| **Any cardiovascular disease** | 735 (0.8) | 241 (0.8) | 1.03 (0.89, 1.20) | 0.99 (0.85, 1.14) |
| **Ischemic heart disease** | 269 (0.3) | 70 (0.2) | 0.82 (0.63, 1.06) | 0.78 (0.60, 1.02) |
| Myocardial infraction | 22 (0.0) | 3 (0) | 0.44 (0.13, 1.47) | 41 (0.12, 1.37) |
| **Cerebrovascular disease** | 215 (0.2) | 64 (0.2) | 0.94 (0.71, 1.24) | 0.89 (0.68, 1.18) |
| **Stroke** | 84 (0.1) | 18 (0.1) | 0.68 (0.41, 1.13) | 0.64 (0.39, 1.07) |
| Ischemic stroke | 59 (0.1) | 11 (0) | 0.59 (0.31, 1.13) | 0.56 (0.29, 1.07) |
| Hemorrhagic stroke | 29 (0.0) | 8 (0) | 0.88 (0.40, 1.92) | 0.83 (0.38, 1.81) |
| **Heart failure** | 61 (0.1) | 25 (0.1) | 1.29 (0.81, 2.06) | 1.23 (0.77, 1.96) |
| Cardiomyopathy | 13 (0) | 5 (0) | 1.21 (0.43, 3.40) | 1.16 (0.41, 3.24) |
| Valvular heart disease | 9 (0) | 1 (0) | 0.35 (0.04, 2.79) | 0.33 (0.04, 2.63) |
| **Arrhythmia** | 252 (0.2) | 94 (0.3) | 1.17 (0.93, 1.49) | 1.13 (0.89, 1.43) |
| Arterial fibrillation | 44 (0) | 10 (0) | 0.72 (0.36, 1.43) | 0.68 (0.34, 1.36) |
| **Venous thromboembolism** |  |  |  |  |
| Deep vein thrombosis | 31 (0) | 18 (0.1) | **1.85 (1.03, 3.30)** | 1.75 (0.98, 3.12) |
| Pulmonary embolism | 7 (0) | 3 (0) | 1.32 (0.34, 5.10) | 1.29 (0.33, 4.98) |
|  |  |  |  |  |
| **Surgery with chemotherapy (N = 7,155)** |  |  |  |  |
| **Any cardiovascular disease** | 331 (0.7) | 99 (1.1) | **1.60 (1.27, 2.01)** | 0.90 (0.72, 1.13) |
| **Ischemic heart disease** | 120 (0.3) | 18 (0.2) | 0.84 (0.51, 1.38) | 0.45 (0.27, 0.73) |
| Myocardial infraction | 1 (0) | 0 (0) | - | - |
| **Cerebrovascular disease** | 103 (0.2) | 19 (0.2) | 1.01 (0.62, 1.65) | 0.55 (0.34, 0.90) |
| **Stroke** | 37 (0.1) | 17 (0.2) | **2.34 (1.30, 4.20)** | 1.39 (0.78, 2.47) |
| Ischemic stroke | 23 (0) | 11 (0.1) | **2.47 (1.19, 5.13)** | 1.45 (0.71, 2.97) |
| Hemorrhagic stroke | 16 (0) | 8 (0.1) | **2.57 (1.09, 6.06)** | 1.51 (0.65, 3.52) |
| **Heart failure** | 33 (0.1) | 12 (0.1) | **2.05 (1.05, 4.00)** | 1.09 (0.56, 2.11) |
| Cardiomyopathy | 6 (0) | 1 (0) | 0.80 (0.09, 6.70) | 0.50 (0.06, 4.15) |
| Valvular heart disease | 2 (0) | 1 (0) | 3.23 (0.29, 35.65) | 1.50 (0.14, 16.68) |
| **Arrhythmia** | 117 (0.3) | 28 (0.3) | 1.29 (0.85, 1.95) | 0.72 (0.47, 1.08) |
| Arterial fibrillation | 19 (0) | 8 (0.1) | 2.13 (0.92, 4.93) | 1.27 (0.55, 2.89) |
| **Venous thromboembolism** |  |  |  |  |
| Deep vein thrombosis | 8 (0) | 25 (0.3) | **15.52 (6.93, 37.74)** | **9.45 (4.27, 20.93)** |
| Pulmonary embolism | 3 (0) | 14 (0.2) | **21.63 (6.14, 76.16)** | **14.06 (4.05, 48.81)** |

*Matching variables: age, sex, income, residential area, and comorbidities (diabetes mellitus, hypertension, and hyperlipidemia)

†Subhazard ratios for events were modeled with mortality as a competing risk.

AYA, adolescent and young adult; CI, confidence interval; HR, hazard ratio.
